# Prevalence and associated risk factors for sexual dysfunction among postmenopausal women: a study from Iran

**DOI:** 10.1186/s40695-021-00069-0

**Published:** 2021-11-27

**Authors:** Azadeh Tavoli, Zahra Tavoli, Mohammad Effatpanah, Ali Montazeri

**Affiliations:** 1 Department of Psychology, Faculty of Education and Psychology, Alzahra University, Tehran, Iran; 2 Department of Obstetrics and Gynecology, School of Medicine, Tehran University of Medical Science, Tehran, Iran; 3 Department of Psychiatry, School of Medicine, Tehran University of Medical Science, Tehran, Iran; 4 Population Health Research Group, Health Metrics Research Centre, Iranian Institute for Health Sciences Research, ACECR, Tehran, Iran; 5 Faculty of Humanity Sciences, University of Science and Culture, Tehran, Iran

**Keywords:** Menopause, Sexual dysfunction, Women, Iran

## Abstract

**Background:**

Female Sexual Dysfunction (FSD) is a distressing condition linked to menopause. This study aimed to determine the prevalence and contributing factors for FSD among postmenopausal women.

**Methods:**

This was a cross-sectional study. A convenience sample of postmenopausal women attending a gynecology clinic in a teaching hospital affiliated with Tehran University of Medical Sciences was enrolled into the study. The Female Sexual Function Index (FSFI) was used to assess sexual function. In addition, demographic and psychosocial information were recorded. The association between sexual function and anxiety and depression were examined to explore the data.

**Results:**

In all 162 postmenopausal women were studied. We performed general linear regression analysis to assess the relationship between sexual function and anxiety while including demographic variables in the model. The results showed that the model could explain about 46% of the variance observed in sexual function (adjusted *R*^2^ = 0.467). The analysis indicated that among independent variables, age (*p* <  0.001), sexual frequency (*p* <  0.001), and anxiety (*p* = 0.003) were significant contributing factors associated with sexual function. A similar analysis evaluating the relationship between sexual function and depression in menopausal women found that age (*p* <  0.001), sexual frequency (*p* <  0.001), and depression (*p* = 0.003), were significant contributing factors associated with sexual function; explaining about 46% of the variance observed (adjusted *R*^2^ = 0.466).

**Conclusion:**

The findings showed that nearly half of menopausal women had sexual dysfunction in this convenience sample of women seeking gynecologic care. Women reporting sexual dysfunction also reported a higher prevalence of anxiety and depression. Indeed, recognition of such factors requires a holistic therapeutic approach to sexual dysfunction among postmenopausal women.

## Background

Female Sexual Dysfunction (FSD) is one of the distressing conditions that affect many women worldwide [1]. Sexual dysfunction is not a single entity but a group of various adverse conditions including low sexual interest, arousal, orgasm, and satisfaction, and pain that leads to sexual dysfunction among women [2]. However, the transition from reproductive age to menopausal status might worsen this condition further, although controversy exists regarding the direct role of menopause on sexual dysfunction [3, 4]. Some investigators showed that sexual dysfunction could affect up to 60% of postmenopausal women [5, 6] most of whom would suffer from decreased sexual desire [7]. It is argued that the change in circulating sex hormones in postmenopausal women may influence emotional and cognitive aspects of sexuality, and thus sexual dysfunction in these women may occur more often [8].

Evidence suggests several factors affect sexual functioning in postmenopausal women. The most relevant variables are age, education, body image, general and mental health, achievement of reproductive goals, self-esteem, norms and experiences while, general and sexual health of the partner, and duration and quality of partnership are important also [9]. It is well documented that emotional distress and depression are independent risk factors for low sexual function in women [10].

Although prevalence and concerns about sexual dysfunction among Iranian women is well reported [11–13], information on sexual functioning among postmenopausal women is scarce. This study aimed to determine the prevalence and risk factors for sexual dysfunction among postmenopausal women. It was hoped that the findings from this study could contribute to the existing literature and help in designing appropriate interventions for this population.

## Methods

### Design and procedure

This was a cross-sectional study to assess sexual dysfunction among a sample of postmenopausal women in Tehran, Iran in 2018. Women who agreed to participate in the study were asked to respond to a questionnaire while they were waiting to visit a gynecologist.

### Participants

The convenience sampling method was applied to recruit a sample of postmenopausal women attending a gynecology clinic in a teaching hospital affiliated with Tehran University of Medical Sciences during one calendar year. All women were attending the clinic either for routine care or other disease conditions. Information on menopausal status was recorded by a gynecologist. It was defined as no menstruation 12 months after the last normal menstrual period (LNMP). All women willing to participate were considered eligible except women using medications that affect sexual function; women who were sexually inactive; and women having a partner diagnosed with sexual dysfunction. The required sample size was estimated using Cochran’s formula (*n* = Z^2^ × pq/d^2^) considering the following parameters: Z = 1.96, *p* = 0.5, q = 0.5, d = 8%. As such we estimated that a study with a sample of 150 menopausal women would have 80% power at 5% significance level to detect sexual dysfunction. However, considering a 10% refusal, we aimed to recruit 165 menopausal women.

### Independent variables

To describe the sample age, age at menopause, body mass index (BMI), sexual frequency, history of non-hormonal medication and gynecological surgery, and family size were recorded. In addition, the Hospital Anxiety and Depression Scale (HADS) was used to assess if women suffered from anxiety or depression or both [14]. The questionnaire contains 14 items where 7 items measure anxiety and 7 items measure depression. The score for each subscale ranges from 0 to 21. Scores of 11 or more on either subscale are considered to be a significant ‘case’ of psychological morbidity, while scores of 8–10 represent ‘borderline case’ and 0–7 are considered ‘normal’ [14]. We used the Iranian version of HADS that has proved to be a valid and reliable measure of anxiety and depression [15].

### Dependent variables

The Female Sexual Function Index (FSFI) was used to measure sexual functioning. It is a 19-item questionnaire, which evaluates six domains of female sexual functioning during the past 4 weeks: desire, arousal, lubrication, orgasm, satisfaction, and pain during sexual intercourse [16]. Each item is scored from 0 to 5. The summed scores of each domain is multiplied by a certain coefficient (desire by 0.6, arousal and lubrication by 0.3, orgasm, satisfaction, and pain by 0.4) for a total score domain. The total FSFI score is the sum of these domain scores, with a minimum of 2 and a maximum of 36. The highest score shows the best sexual function and less pain [14]. The psychometric properties of the Iranian version of the FSFI are well documented [17].

### Statistical analysis

Descriptive statistics including frequencies, means and standard deviations were used to explore the data. We used general linear regression analysis to evaluate the relationship between sexual function and independent variables including age, BMI, sexual frequency, anxiety, and depression. The selection of independent variables was based on findings from previous investigations. It has been shown that age, BMI, sexual frequency, anxiety, and depression could have a significant effect on the sexual function of postmenopausal women [18–21]. Of these, although the inclusion of sexual frequency in the model seems somewhat teleological (e.g. reduced arousal/desire so reduced frequency), evidence suggests that the frequency of sexual intercourse independently might play an important role in improved or decreased sexual function in postmenopausal women [22, 23]. However, due to the small sample size, we used a parsimonious mode. *P*-values less than 0.05 were considered statistically significant.

## Results

In all, 165 menopausal women were approached and 162 women agreed to participate in the study. Three women refused to take part in the study due to dislike. The mean age (SD) of participant was 55.5 (6.16) ranging from 40 to 76 years. Thirty-five women (21.6%) received gynecological surgery. Many participants reported severe anxiety (59.9%) and depression (44.4%). Women’s characteristics including age, body mass index, frequency of sexual activity, history of surgery, medication, anxiety, and depression status are presented in Table 1.

**Table 1:** Clinical and demographic characteristics of 162 menopausal women seeking gynecological care: Tehran, Iran, 2018

**Age in years**	Mean (SD)	55.5 (6.16)
**Age at menopause in years**	Mean (SD)	47.3 (5.85)
**Time since menopause (based on last normal menstrual period in years)**	Mean (SD)	8.1 (7.6)
**Body mass index**	Mean (SD)	28.9 (4.73)
**Frequency of sexual activity per month**	Mean (SD)	1.80 (1.37)
**Non-hormonal medication (No. %)**	Yes	56 (34.6)
	No	106 (65.4)
**Received gynecological surgery (No. %)**	Yes	35 (21.6)
	No	127 (78.4)
**Family size (No. %)**	2	51 (31.5)
	3	38 (23.5)
	4	22 (13.5)
	> 4	51 (31.5)
		**No. (%)**
**Anxiety (score)**	Normal (0–7)	23 (14.2)
	Borderline (8–10)	42 (25.9)
	Severe (11–21)	97 (59.9)
	Mean (SD)	11.2 (4.02)
	Range	1–19
		**No. (%)**
**Depression (score)**	Normal (0–7)	33 (20.3)
	Borderline (8–10)	57 (35.2)
	Severe (11–21)	72 (44.4)
	Mean (SD)	9.97 (4.21)
	Range	0–20

The mean sexual function score was 14.1 (SD = 8.13) out of 36.0. The results for sexual function and its sub-scales are shown in Table 2. The highest score was observed for the ‘pain’ sub-scale indicating less pain (mean = 3.04, SD = 2.18), and the lowest score was reported for ‘arousal’ indicating lower arousal in this sample (mean = 1.90, SD = 1.32).

**Table 2:** Mean and standard deviation (SD) on the female sexual function index^a^ among 162 menopausal women seeking gynecological care: Tehran, Iran, 2018

	Mean	SD
**Desire**	2.01	0.99
**Arousal**	1.90	1.32
**Lubrication**	2.13	2.13
**Orgasm**	2.23	2.25
**Satisfaction**	2.82	2.82
**Pain**	3.04	2.18
**Total score (range)**	14.1 (2.30–28.50)	8.13

^a^Higher scores indicate better conditions

We performed a general linear regression analysis to assess the relationship between sexual function and anxiety including the demographic variables in the model (Table 3). The results showed that the model could explain about 46% of the variance observed in sexual function (adjusted *R*^2^ = 0.467). The analysis indicated that among independent variables, age (*p* < 0.001), sexual frequency (*p* = < 0.001), and anxiety (*p* = 0.003) were significant contributing factors associated with sexual function. The findings indicated that older age, decreased sexual frequency and increased anxiety significantly contributed to decreased sexual function.

**Table 3:** General linear regression model for the association between anxiety and female sexual function index scores (*n* = 162): Tehran, Iran, 2018

	Beta	Type III Sum of Squares	df	Mean Square	F statistic	***P***-value
**Age**	−0.314	589.745	1	589.745	16.651	< 0.001
**BMI**	−0.117	47.091	1	47.091	1.330	0.864
**Sex frequency**	3.490	3544.84	1	3544.84	100.065	< 0.001
**Anxiety**	−0.37	334.013	1	334.013	9.431	0.003

*R* squared = 0.481 (Adjusted *R* squared = 0.467)

Similarly, when performing the analysis evaluating the relationship between sexual function and depression in postmenopausal women we found that age (*p* < 0.001), sexual frequency (*p* < 0.001), and depression (*p* = 0.003) were significant contributing factors associated with sexual function; explaining about 46% of the variance observed (adjusted *R*^2^ = 0.466) (Table 4). Again, the findings indicated that older age, decreased sexual frequency and increased depression significantly contributed to decreased sexual function.

**Table 4:** General linear regression model for the association between depression and female sexual function index scores (*n* = 162): Tehran, Iran, 2018

	Beta	Type III Sum of Squares	df	Mean Square	F statistic	***P***-value
**Age**	−0.382	108.090	1	108.090	3.216	0.075
**BMI**	−0.031	0.416	1	0.416	0.012	0.912
**Sex frequency**	3.947	519.765	1	519.765	15.463	< 0.0001
**Depression**	−3.982	250.459	1	250.459	7.451	0.007

*R* Squared = 0.479 (Adjusted *R* Squared = 0.466)

## Discussion

The findings from the current study showed that sexual dysfunction among postmenopausal women was associated with age, anxiety and depression. This observation could perhaps be explained in part by the fact that almost all women at this stage of life are experiencing changes in hormonal and physiological attributes that affect their sexual function and sexuality [24]. Additionally, other contributing factors might influence sexual dysfunction in this population.

The current study showed that anxiety and depression significantly were associated with sexual dysfunction. The association between psychological factors and sexual dysfunction are complicated and might be explained by the fact that postmenopausal women experience lower self-esteem, and weaker body image where both factors lead to reduced sexual desire. In fact, self-esteem and body image contribute to anxiety and depression and these in turn lead to less sexual function [9]. On the other hand, one might argue that less sexual functioning in postmenopausal women by itself might cause anxiety and depression and thus there is a circular pathway between psychological factors and sexual dysfunction in women who experience menopause. Furthermore, some women feel less sexually attractive since they tend to gain weight as their metabolism slows down. For some women, this weight gain harms body image and self-esteem and may lead to mild to moderate depression [25].

The findings showed that problems in arousal was the most common sexual dysfunction reported in these postmenopausal women while other studies found that desire [26], lubrication, dyspareunia, and arousal [27, 28] were the most common sexual problems among postmenopausal women. Perhaps the differences observed between the affected domains of sexual dysfunction in various studies might be due to variations in life style and level of physical activity and other cultural and socioeconomic factors [29–31].

We found that sexual frequency, anxiety, and depression independently associated with sexual function. These findings are in line with other studies on the topic [4, 32]. The relationship between mental health and sexual dysfunction is well documented [33]. Previous research on the predictive role of demographic factors in sexual problems has shown a negative association between age and sexual function. Some studies have even found that a spouse’s age is a very influential factor in women’s sexual function [34, 35]. However, the differences in sexual dysfunction in older women might be due to the different research methods, the measures used in various studies, different cut-off points selected in the studies, different living condition, racial, religious, and cultural and attitudinal factors [36–39].

The relationship between moderate/severe sexual dysfunction with non-regular weekly intercourse has been previously documented in women of all ages [4]. It warrants further interventional research inquiring into whether encouraging more intercourses can improve the sexual function of postmenopausal women. It seems quite reasonable to consider that non-regular and unsatisfactory sexual intercourses act as a vicious cycle since the less frequently postmenopausal woman has intercourses, the more likely she will suffer sexual dysfunction. One might argue that a majority of these women avoid more sexual intercourse due to pain, feeling unwanted, being anxious, and being uneducated about lubrication and how to have better intercourse, although the current study did not describe such observation. Altogether, we know that FSD is a well-known problem in postmenopausal women due to its multi-facet psycho-socio-biological nature, which necessitates a holistic approach [7, 32, 40, 41].

Sexual function components may be affected by menopause due to changes in sensory perception, central and peripheral nerves, peripheral blood flow, and the capacity to create muscle tension in response to estrogen deficiency. These changes may lead to lower self-esteem, weaker body image, and reduced sexual desire. Some women feel less sexually attractive or less satisfied with their bodies. In fact, their self-image can change because they tend to gain weight as they age and their metabolism slows down. Thus, depression and emotional distress, in turn, can be a risk factor for sexual dysfunction [22, 42], and it can be imagined a mutual relationship between emotional state and sexual function.

The present study had some limitations. This was a cross-sectional study and thus the findings should be interpreted with caution. Secondly, we could not measure the hormonal status of the participants and therefore the clinical application of the study is limited. We suggest conducting interventional studies for changing those modifiable risk factors associated with FSD in postmenopausal women.

## Conclusion

The findings showed that nearly half of menopausal women had sexual dysfunction in this convenience sample of women seeking gynecologic care. Those who were noted to have sexual dysfunction also reported a higher prevalence of anxiety and depression. Indeed, recognition of such factors requires a holistic therapeutic approach to sexual dysfunction among postmenopausal women.

## Data Availability

The data used and analyzed for writing this manuscript are available on request from the corresponding authors.

## Abbreviations

BMI: Body mass index
FSD: Female Sexual Dysfunction
FSFI: Female Sexual Function Index
HADS: Hospital Anxiety and Depression Scale
